# Body size awareness matters when dogs decide whether to detour an obstacle or opt for a shortcut

**DOI:** 10.1038/s41598-023-45241-w

**Published:** 2023-10-19

**Authors:** Péter Pongrácz, Petra Dobos, Tamás Faragó, Enikő Kubinyi, Rita Lenkei

**Affiliations:** 1 Department of Ethology, Institute of Biology, Eötvös Loránd University, Pázmány Péter Sétány 1/C, 1117 Budapest, Hungary; 2 MTA-ELTE Lendület “Momentum” Companion Animal Research Group, Budapest, Hungary; 3 Department of Ethology, Neuroethology of Communication Lab, Budapest, Hungary; 4 Doctoral School of Biology, Institute of Biology, ELTE Eötvös Loránd University, Budapest, Hungary

**Keywords:** Ecology, Neuroscience

## Abstract

Body-awareness is one of the fundamental modules of self-representation. We investigated how body-awareness could contribute to dogs' decision making in a novel spatial problem where multiple solutions are possible. Family dogs (N = 68) had to obtain a treat from behind a transparent fence. They had two options: either detour around the fence (7 m), or take a shortcut through a doorway (2 m). We had three conditions: small door open, large door open, and doors closed. Our results indicated that dogs assess the size of the doorway, and if they find it too small, they decide to detour instead, while in the case of the open large door, they rather opted for the shortcut without hesitation. Shorter headed dogs tended to choose open doors more often, while longer headed dogs rather chose detours, probably because of their better peripheral vision. While body size awareness did not manifest differently in dogs with short or long heads, we showed for the first time a connection between head shape and physical cognition in dogs. We showed that dogs rely on their body-awareness in a naturalistic setting where multiple solutions exist simultaneously. Dogs make decisions without lengthy trial-and-error learning and choose between options based on their body-awareness.

## Introduction

Self-representation, in both its complex entirety^1^, and its development during ontogeny^2^, is one of the classic cognitive concepts in human psychology and comparative cognitive sciences. The strong connection between human-centric cognitive research and the core concept of the 'one and undividable' self-representation^3^, has set the course for comparative research, even in non-human species, for a long time. Although there is a growing body of evidence that at least simpler forms of self-representation may exist in most of the non-human species^4^, researchers still prefer to focus on the 'higher end' (most complex) forms of self-recognition that would develop even in humans, towards the later stages of their cognitive development^5,6^. From an ethological perspective, the main problem with this top-down approach is the lack of ecological validity, in other words, by searching for higher-end cognitive attributes in non-human species, we can rarely assess the function (adaptivity) of this feature in the given species^7^.

Recently, we suggested a new, modular framework^8^ that allows researchers to investigate the components of self-representation separately. These modules can be regarded as loosely connected cognitive capacities of the given species, and the presence and relative importance of one or another module, depends on the specific adaptation processes of that species. The modules range from the simplest forms of sensorimotor interference that provide the necessary feedback for differentiating the self from the environment^9^, through various forms of body-awareness (e.g.,^10^), to more complex cognitive features such as episodic-like memory (e.g.,^11^), empathy (e.g.,^12^) and the formation of theory of mind (e.g.,^13^).

Body-awareness is regarded as one of the fundamental building blocks of self-representation^8,14^, as it provides the basic capacity to distinguish between the self and the environment, through various physical interactions between them. During navigation in the physical environment, having information about the body's extension and other physical features has definite advantages and it is thought to be the phylogenetically earliest appearance of any kind of self-knowledge^15^. The process of its development^16^, and the factors that influence it^17^, have been described in detail in humans. Body-awareness develops through learning from various interactions with the environment, and it is basically a capacity of the individual to store and retrieve information about its body^18^, as an existing physical object that also has a distinct size (i.e., body-size awareness,^8^).

Body-size awareness allows the individual to negotiate its way amid physical obstacles by comparing its size to the various gaps, openings, and straits in the environment. Obviously, this mental modeling of one's own size could be replaced with other mechanisms, such as trial-and-error attempts, learning the exact nature of every obstacle, or avoiding any confinement. However, it is easy to assume that the mental capacity to rely on one's own size provides the maximum level of flexibility, which, in theory, would be a highly adaptive trait in every species characterized by a relatively large body size, fast locomotion, and existence in a complex environment^10^.

There are only a handful of experiments that study body-awareness in non-human animals, though this capacity is crucial for flying animals, since collision with obstacles could cause injuries or death. Moreover, as some birds are very fast fliers and they live in complex environments, they have only split seconds to decide whether a gap is large enough for them or not. For them, it is pivotal to be aware of the size of their wingspan relative to the width of a particular gap. Indeed, it was found that budgerigars (*Melopsittacus undulatus),* can accurately estimate a gap's width and alter their movement when its size falls within 6% of their wingspan^19^. Negotiating narrow gaps while flying was also tested in bumblebees (*Bombus terrestris*). These eusocial insects are excellent subjects for body-size awareness experiments because workers of different tasks greatly vary in their body size and they fly in dense vegetation. As in the case of birds, they also adjusted their behavior according to their own size^20,21^. Experiments connected to body-awareness are also intriguing in species where body size of the individual can rapidly change over time. A door choice task was applied to rat snakes (*Elaphe radiata*), whose body diameter considerably increases after feeding. It was shown that they can learn to choose the adequately sized opening to reach a shelter^22^.

Investigation of body-awareness in dogs is particularly interesting because dogs are not only a cognitively resourceful and fast-moving terrestrial mammal living in complex human environments, but also the most variable species on Earth in terms of their physical features due to artificial selection for different working and aesthetic purposes^23^. For instance, based on breed standards, there is approximately a 30-fold difference in weight between a Chihuahua and an Irish wolfhound. This means that knowledge about their body size cannot be genetically hard-wired in dogs, suggesting the need for at least some kind of behavioral flexibility when negotiating challenging environmental obstacles. As body size has been found to play an important role in dogs during social interactions, such as in agonistic encounters (e.g., indexical information encoded in agonistic dog growls;^24–26^), it would be reasonable to assume that dogs possess at least rudimentary knowledge about their own size.

Regarding body-awareness in dogs, using the modified 'body as an obstacle' paradigm that was originally developed to test human toddlers^18^, we showed that similar to humans and Asian elephants (*Elephas maximus*)^27^, dogs also recognize if their body is an obstacle during a problem-solving task, consequently they understand the connection between their body and the environment^10^. In another study, in a series of door choice tasks, we found that dogs approached 'too small' openings with significantly longer latencies than 'large enough' openings. Our results suggested that dogs have an expectation of whether they would be able to pass through an opening or not. It is important to note that in this task, the dogs did not have the opportunity to choose the more preferred opening, and thus they had to make an a priori decision based not only on external cues, but also on their body size awareness^8^. Horowitz and colleagues have also described in detail how dogs adjust their behavior and movement patterns in response to apertures that are smaller than their body height^28^.

In the previous experiments, only one opening was present at a time for the subjects, therefore dogs approached regardless of whether it was big enough for them to go through or not, though their performance was slower if they later decided not to go through. In ecologically valid scenarios, which are the cornerstone of ethology^29^, animals often face several solutions to the same problem. Therefore, in this experiment, our goal was to obtain a more detailed understanding of how dogs incorporate body-awareness into their decision-making process when faced with a novel spatial problem task. More importantly, the solution that requires knowledge of their own size would be only one of several possibilities.

Our main question was, would dogs rely on their knowledge of their own size when facing a problem with multiple solutions, where only one solution requires body awareness? To address this, we developed a paradigm where, in addition to the opening that was either too small or large enough to pass through, we offered an alternative solution (i.e.: detouring the obstacle), to solve the problem (i.e., reach the reward on the other side of the fence). Based on our earlier research, we knew that while dogs can master detours around a transparent fence, this task has a considerable level of difficulty for them^30,31^. We hypothesized that the dogs' choice would be affected by both their body-awareness (when deciding whether an opening is large enough or not), and the relative difficulty of the two solutions (when a parallel, suitably sized opening, and the detour are offered). We expected that dogs would only choose the detour if they recognized that the shortcut to the target (the opening) was too small for them. However, when the opening was large enough, we expected that, based on their knowledge about their body size, dogs would choose the shortcut over the more laborious detour. While solving the problem, another important parameter is how quickly the dogs approach the openings or perform the detour. We hypothesized that the latency of approaching the doors or making a detour would be in association with the difficulty of the task, in other words, it would show whether dogs hesitated more or less during decision making. We predicted that the latency to detour would be shorter when the dogs notice that the door was too small for them. At the same time, we predicted that the dogs would approach the large open door with shorter latencies than the small one. Another parameter that can serve as indicator of the difficulty of the task was the looking at the humans. Earlier it was found that when dogs struggled more with making a detour around a V-shaped fence they looked at their owners more frequently^30^. Here we predicted that dogs will look at the nearby humans (experimenter, owner) more often if they face a more difficult to solve problem: a small door or the need for a detour versus the easy shortcut through the large door.

An additional factor whose potential effect we investigated, was the head shape of the dogs (as expressed in the cephalic index—CI). Earlier studies have found that brachy- and dolichocephalic dogs perform differently in tasks where visual perception is important^32,33^. However, in those studies, dogs were tested in social tasks, where interspecific interactions, such as following human communicative signals, were the focus. In our present study, dogs had to overcome an obstacle on their own based on visual perception (i.e., finding the optimal solution around or across a barrier). Importantly, during the task in our current study, although the experimenter and the dog’s owner were present, they had to refrain from providing any directional cues to the dog. McGreevy et al.^34,35^, have shown that there are differences in the retinal and brain anatomy of brachy- and dolichocephalic dogs, thus we hypothesized that the cephalic index of the subjects may show associations with the speed of decision-making and the ratio of erroneous/suboptimal decisions.

## Results

Due to the complex nature of the statistical analyses, we briefly summarize here the order of results as they will be presented in this chapter. The results are divided into two main sections: first we discuss those results that did not involve the significant effect of dogs’ CI; then we present those results that show a significant association with the CI. Within each of these sections, the results are presented in the following order: choice of solution type (door or detour); latencies of detour; latencies of approaching the door; looking frequencies.

**Figure 1:**
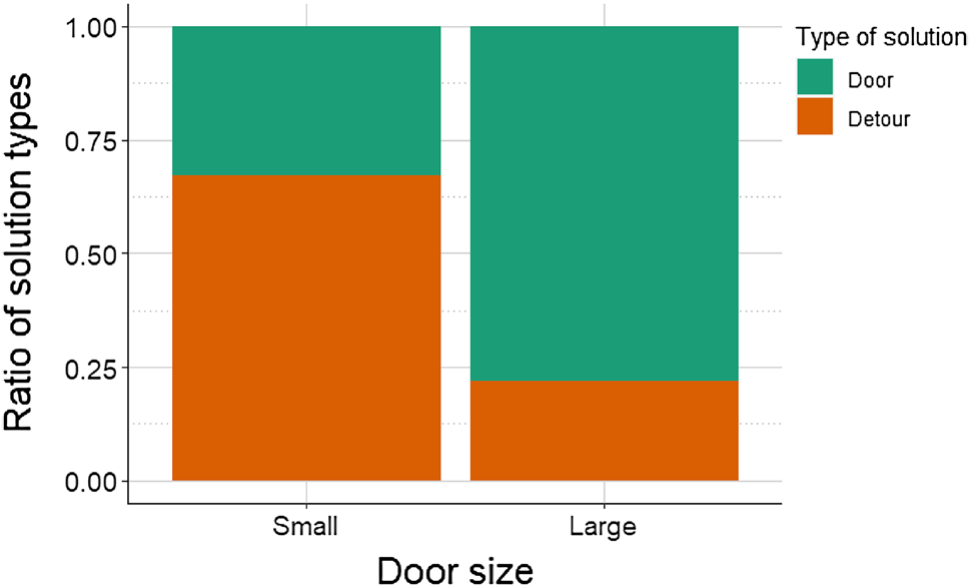
Proportion of dogs choosing to make a detour around the fence or approaching the door, when the small or large door was open.

At first, we analyzed whether dogs would choose the detour or the door in the first trial where both options were available (i.e., either the small or the large door was open). According to the results, the type of solution (detour or door) was only affected by the door size (LR test: *χ*^2^(1) = 70.58; *p* < 0.001; GLMM: β ± SE = − 20.572 ± 2.919 ; z = − 7.047; p < 0.001). If the small door was open, significantly more dogs opted for the detour (see Fig. 1). More importantly, the number of closed-door trials (1 vs. 3 detours at the beginning of the session in Group 2–3 vs Group 1) did not affect the type of solution (LR test: *χ*^2^(2) = 0.220; *p* = 0.896) when dogs encountered an open small or large door for the first time. In other words, door-naive dogs (i.e., those who had not encountered any open doors before) did not show stronger preference for making a detour if they had more a priori experience with detours. The height of the dogs did not show a significant association with the type of solution (door or detour), neither in relation to the door size (LR test: *χ*^2^(1) = 0.007; *p* = 0.935) nor in general (LR test: *χ*^2^(2) = 0.114; *p* = 0.736).

We also analyzed the detour latencies in relation to the repeated exposure of dogs to small or large open doors (see Supplementary Fig. S1). We found a significant interaction between the trial number and the door size (LR test: *χ*^2^(2) = 7.183; *p* = 0.028). Dogs were quicker to make the detour when encountering the open small door for the third time compared to the first small door trial (exp*(β)* [95% CI] 0.609[0.391–0.951]; *z* = − 2.609; *p* = 0.025). However, we did not find any significant differences in detour latencies in the case of the large open door—dogs performed detours with similar latencies across these trials (note that the proportion of dogs that opted for detour in the case of the large open door was relatively low, Fig. 1).

**Figure 2:**
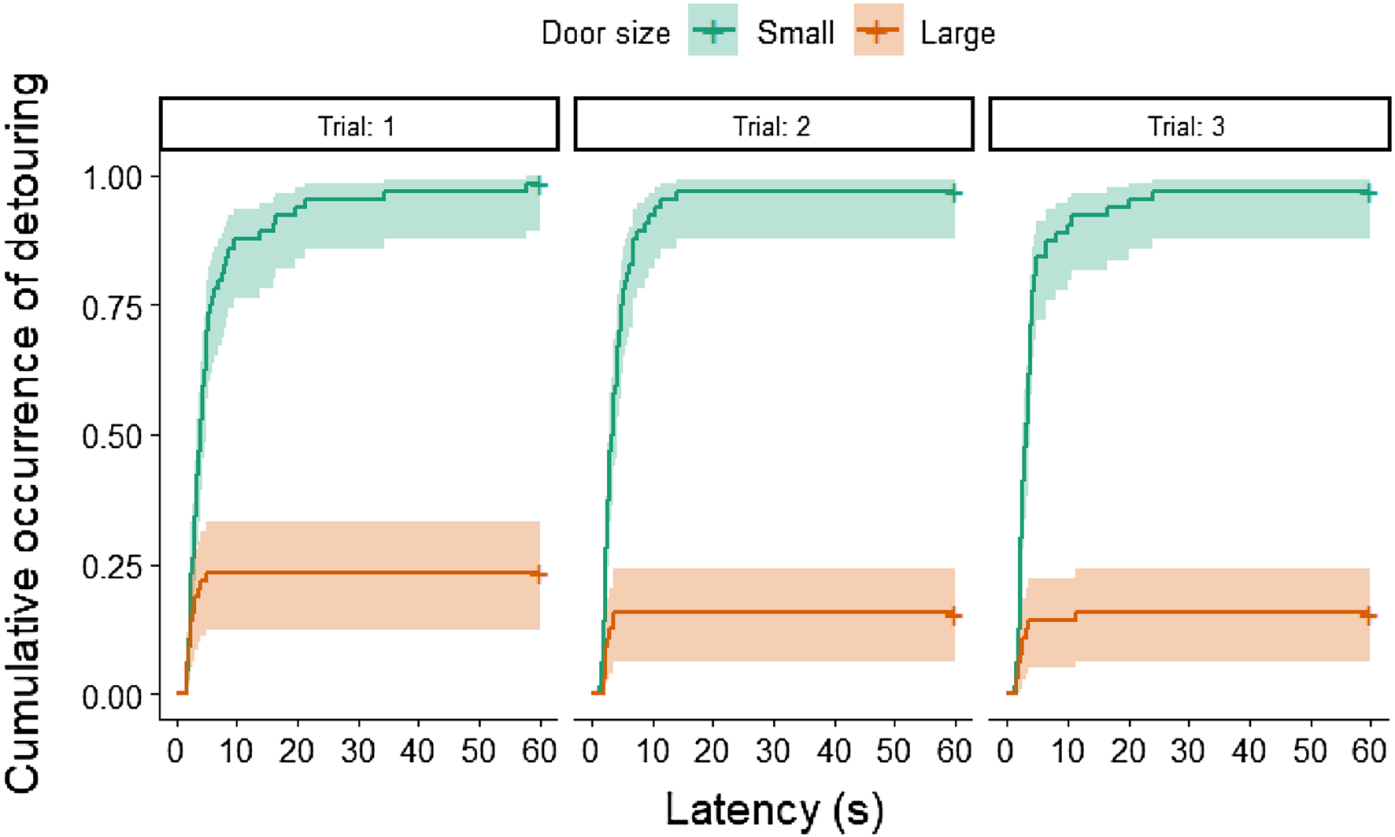
Proportion of dogs at a given latency who performed the detour around the fence, Trial by Trial, when the small or the large door was open. Note that Trial 1, Trial 2 and Trial 3 is a relative designation here, it refers to those trials when in a given group the small or large door was open first, second and third time. Shading ± 95% CI.

We also compared the detour latencies, trial by trial, between the experimental groups (i.e., when the large or the small door was open). We found that dogs performed the detour faster in each trial with the small door open (see Fig. 2) compared to the trials with the large open door (1st Trial with door open: exp*(β)* [95% CI] 11.2[6.11–20.5]; *z* = 7.84, *p* < 0.001; 2nd Trial with door open: exp*(β)* [95% CI] 27.0[13.17–55.4]; *z* = 8.99, *p* < 0.001; 3rd Trial with door open: exp*(β)* [95% CI] 36.4[17.65–75.2]; *z* = 9.72, *p* < 0.001).

**Figure 3:**
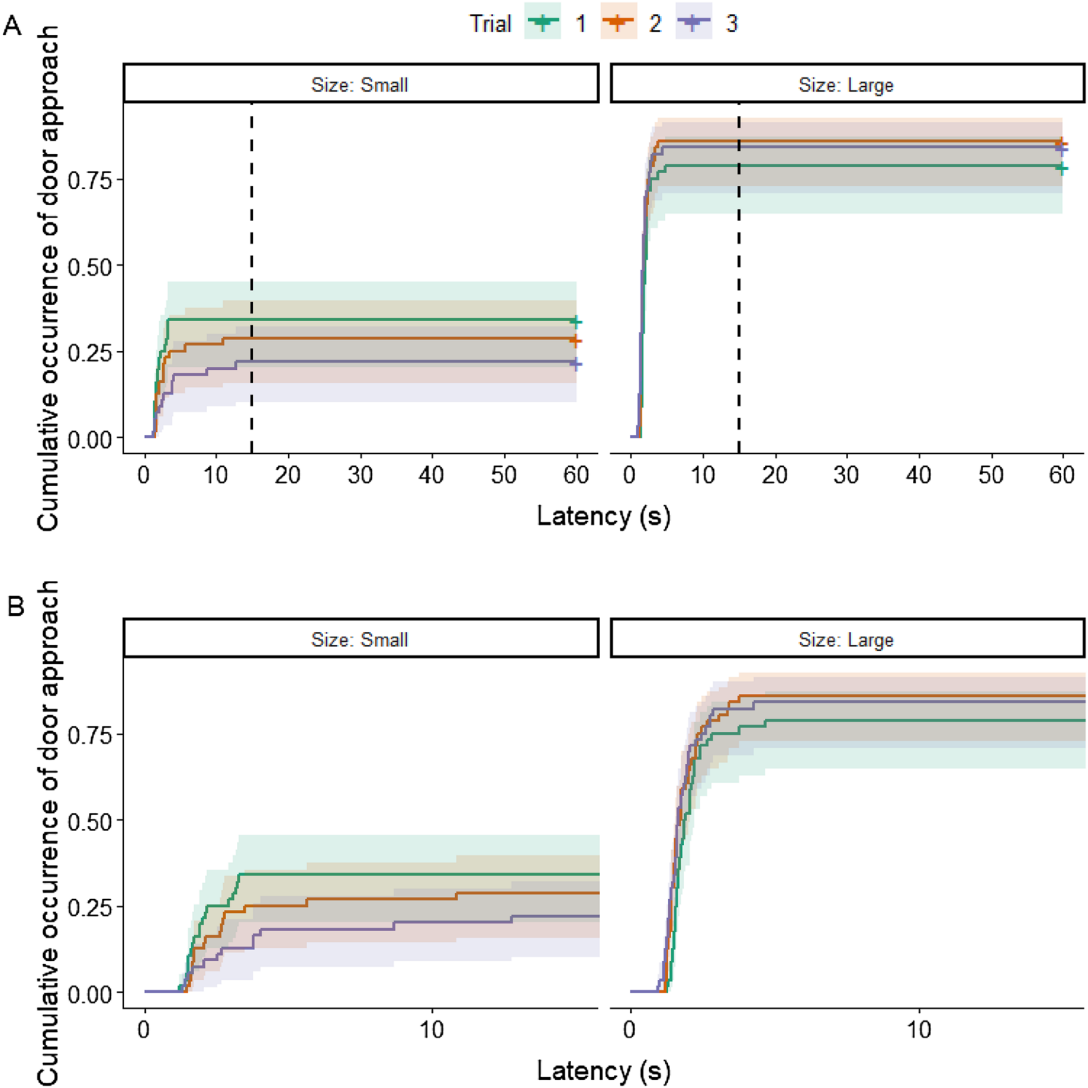
Proportion of dogs at a given latency who approached the door, in the case of the three trials with an open small or large door. (**A**) shows the full data, (**B**) shows the same but censored at 15 s for better visibility. Shading: ± 95% CI.

In the case of the latency of approaching the door, we found a significant interaction between the trial number and door size (*χ*^2^(2) = 9.19; *p* = 0.01). In the case of the large door, dogs approached the door sooner in the second Trial (exp*(β)* [95% CI] 0.577 [0.347–0.960]; *z* = − 2.532; *p* = 0.031) and third Trial (exp*(β) *[95% CI] 0.586 [0.349–0.984]; *z* = − 2.417, *p* = 0.041) than in the first Trial with the open large door. Dogs rarely approached the small open door, and their latencies did not differ among the trials (see Fig. 3).

**Figure 4:**
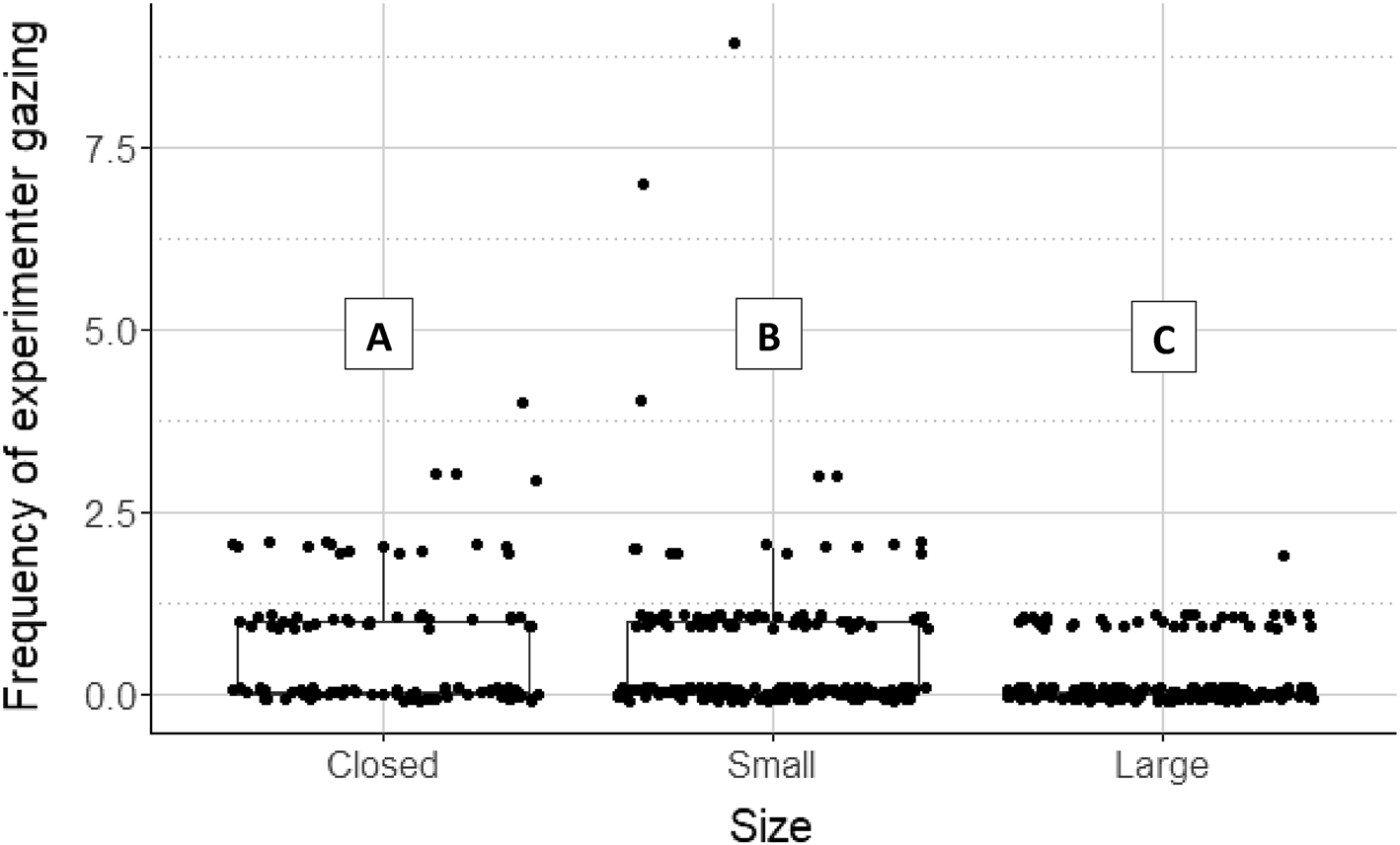
Frequency of looking at the experimenter. Note that the ’groups’ on the horizontal axis are not the original testing groups but they were defined on the basis of the state of the door (closed = no open door; small = the small door is open; large = the large door is open). Different letters above the boxplots indicate significant between-group differences.

When dogs look at a nearby human during a problem-solving task, it is often connected to the task's difficulty level, such as the detour around an obstacle task^30,36^. In the case of looking at the Experimenter, our results showed a significant association with the door size (including also whether any door was open or not) (LR test: *χ*^2^(2) = 54.80; *p* < 0.001). Dogs looked at the Experimenter most often when the doors were closed (closed vs small: *β* ± SE = 0.498 ± 0.160; *z* = 3.117; *p* = 0.005; vs large: *β* ± SE = 1.413 ± 0.203; *z* = 6.962; *p* < 0.001), the next most often looking back occurred when the small door was open (small vs large: *β* ± SE = 0.915 ± 0.192; *z* = 4.759; *p* < 0.001). The frequency of looking at the Experimenter was lowest when the large door was open (see Fig. 4).

We found a similar result in the case of the frequency of looking at the Owner (see Supplementary Fig. S2). This parameter also showed a significant association with the door size (LR test: *χ*^2^(2) = 24.861; *p* < 0.001). As in the case of the Experimenter, dogs looked at the Owner most often in the case of the closed door (closed vs small: *β* ± SE = 0.788 ± 0.287; *z* = 2.747; *p* = 0.017; vs large: *β* ± SE = 1.774 ± 0.389; *z* = 4.557; *p* < 0.001), then the next most often was in the case of the open small door (small vs large: *β* ± SE = 0.987 ± 0.391; *z* = 2.525; *p* = 0.031), and finally, least often when the large door was open.

Dogs also looked significantly more often at the Experimenter than at the Owner (LR test: *χ*^2^(1) = 68.413; *p* < 0.001).

The frequency of looking at the door (see Supplementary Fig. S3) was in association with the door size, including the option when no door was open as well (LR test: *χ*^2^(2) = 16.69; *p* < 0.001). Dogs looked at the large open door the least often, compared to the frequencies of looking at the open small door and towards the closed doors (closed vs small: *β* ± SE = 0.181 ± 0.105; *z* = 1.724; *p* = 0.196; vs large: *β* ± SE = 0.442 ± 0.111; *z* = 3.986; *p* < 0.001; small vs large: *β* ± SE = 0.260 ± 0.098; *z* = 2.666; *p* = 0.021).

### Results regarding association with the cephalic index (CI) of dogs

In the case of door choice, in the trials where dogs first time encountered the large or small door, we found a significant negative effect of CI (exp*(β)* [95% CI] 0.002 [0.000–0.144; z = − 2.890], *p* = 0.004). According to this, dogs with high CI (shorter heads) approached the door significantly more often than the longer headed dogs did, who opted more often for the detour right at the beginning of the trials.

**Figure 5:**
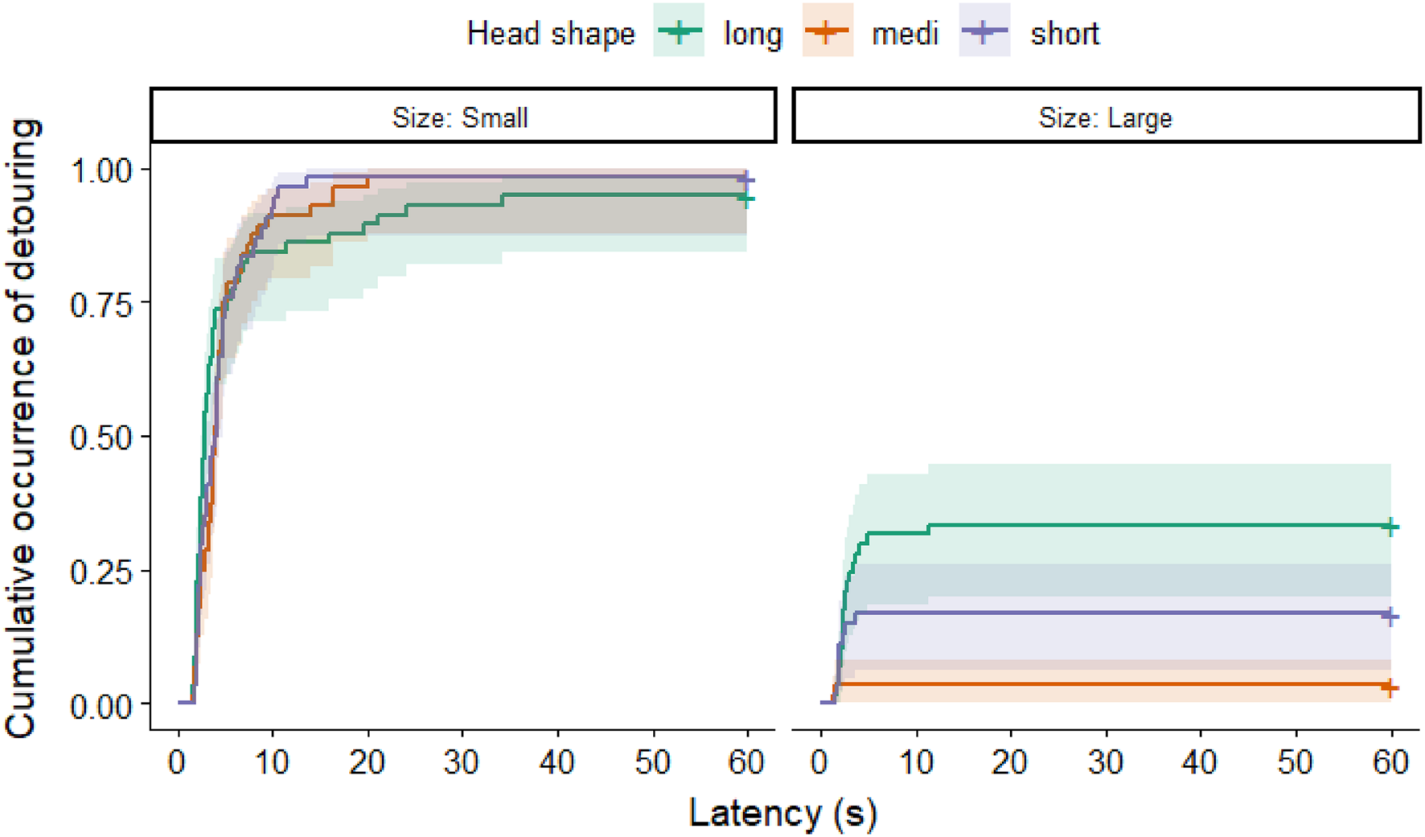
Proportion of dogs at a given latency, who performed a detour in the case of the open small or large door. In order to visualize the results, the otherwise continuous CI score was divided at the 33rd and 67th percentiles. Different colors mark different lengths of head. Shading: ± 95% CI.

Regarding the detour latencies, we found a significant interaction between the CI of dogs and door size (LR test: *χ*^2^(1) = 5.581; *p* = 0.015). In the case of the large door, longer headed dogs that opted for the detour, performed it faster than the shorter headed dogs did (small: exp*(β)* [95% CI] 0.997 [0.958–1.038]; large: exp*(β)* [95% CI] 0.921 [0.855–0.991] Fig. 5). It is important to see that the shorter headed dogs were of the same height in our sample as the longer headed dogs were, thus the difference in their detour latencies was not the consequence of the different length of their legs or general speed of locomotion. As CI was not associated with detour latencies in the case of the closed door trials, we can confidently say that shorter headed dogs were not slower in general, than the longer headed dogs.

**Figure 6:**
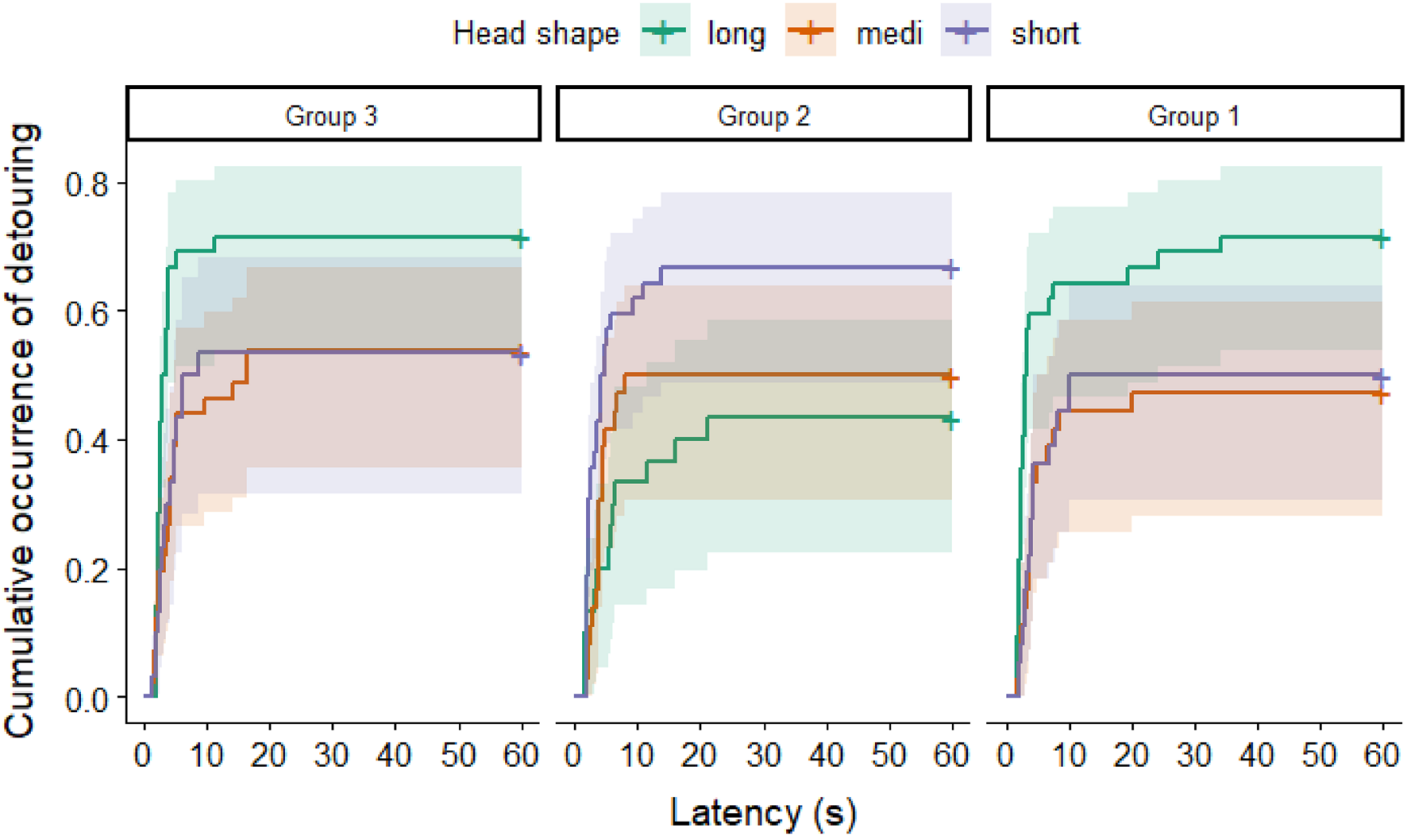
Proportion of dogs at a given latency, who performed a detour in the case of the open small or large door, in the three Groups, respectively. Designations of the groups indicate how many trials the dogs had to perform with closed doors and then which door was open in the next trial. Group 1 = three closed door trials, then large door is open; Group 2 = one closed door trial, then large door is open; Group 3 = one closed door trial, then small door is open. In order to visualize the results, the otherwise continuous CI score was divided at the 33rd and 67th percentiles. Different colors mark different lengths of the head. Shading: ± 95% CI.

In the case of detour latencies, the CI also showed a trend-like interaction with the number of closed door trials before the first open door trial (LR test: *χ*^2^(2) = 5.668; *p* = 0.059). The following groups were formed based on the number of closed door trials and the size of the door that was opened afterwards: Group 1 = three closed door trials, then large door is open; Group 2 = one closed door trial, then large door is open; Group 3 = one closed door trial, then small door is open. Examining Fig. 6, a similar pattern can be observed in Groups 1 and 3. Long headed dogs performed the detour significantly faster in Group 1, than the short headed dogs did when three closed door trials preceded the opening of the small door (Group 1: exp*(β)* [95% CI] 0.905 [0.840–0.975]). In the other two groups, the trend was not significant (Group 2: exp*(β)* [95% CI] 1.021 [0.945–1.102]; Group 3: exp*(β)* [95% CI] 0.952 [0.890–1.019]).

We found a significant effect of CI on the latency of looking at the door (Lr test: *χ*^2^(1) = 4.099; *p* = 0.043). Dogs with a shorter head oriented at the door more often and with shorter latencies than longer headed dogs did, regardless of the door size (exp*(β)* [95% CI] 1.050 [0.998–1.105]; *z* = 1.88, *p* = 0.06; see Supplementary Fig. S4).

Finally, regarding looking at the Experimenter, we found an interaction between the number of closed door trials and CI (LR test: *χ*^2^(2) = 8.145; *p* = 0.017). Shorter headed dogs looked more often at the Experimenter in Group 3, where the small open door was preceded by only one closed door trial (exp*(β)* [95% CI] 2.149[1.249–3.698]).

## Discussion

In our experiment, we offered the dogs a problem-solving task where they had to master a transparent fence that served as an obstacle between the dog and the treat. As we intended to mimic a more natural scenario than the commonly used tests for body-awareness (e.g.,^8^), we provided two alternative solutions for the dog, where only one of them involved a decision based on body-awareness (going through a door which was too small in some of the trials). The other solution was a longer, but steadily available detour around the fence^37^. Our main results showed that dogs indeed relied on their body-size awareness capacity when both an open door and the detour option were available, because they chose the large door (ideal shortcut) over the detour (suboptimal solution), and they opted for the detour when the too small door (erroneous choice) was open. This pattern was also recognizable in the latencies to detour and approach the doors. The height of the dog did not have any association with the dogs' behavior during problem-solving. This was the expected response, as door sizes were established based on the results of^8^, to be either safely big enough (large door), or too small (small door), for each participant. Looking at the humans assisting in the tests (owner and experimenter), supported the theory that the more difficult a task is, the more likely dogs will rely on humans.

According to our main hypothesis, if dogs rely on their body size representation, they would prefer the shortcut over the detour, but only if the door was large enough for them. Here the alternative hypothesis suggested that dogs will always prefer the shorter route, thus they will at first approach the door, regardless of its size. The results confirmed the first hypothesis: *this is the first empirical evidence that dogs use their body representation in a spatial task where they could choose from more than one solution*. We can assume that when dogs face for the first time the open door, they assess it according to their representation about their own size, then decide whether to go straight ahead and do the shortcut through the door or rather perform a (longer) detour. This result supports the finding of^8^. However, at this time, dogs were not 'forced' to try the only available door if they wanted to get to the other side of the obstacle, because they always had the opportunity to make a detour. Approaching the small open door can be considered an erroneous solution, while opting for the detour is the optimal one in this case. The association found between the choices of solution with the CI of dogs can be explained on the basis of the different visual capacity of the brachycephalic and dolichocephalic dogs. The retina of brachycephalic dogs contains a well-defined *area centralis* (with high ganglion density), while dolichocephalic dogs' eyes are characterized by horizontal visual streaks of corresponding high ganglion densities^34^. The difference in their visual neuroanatomy is most probably associated with the distinct performance of short- and long-headed dogs in such tasks where they are required to process visual stimuli that is directly in front of them. Earlier results showed that brachycephalic dogs performed better in two-way pointing experiments^33^, and they established eye contact with humans easier^32^. In our test, short headed dogs may likely have problems with quick assessment of the surrounding environment, however, it is easier for them to focus on the target in front of them. This may cause them to more readily approach and examine even the small door, than the longer headed dogs did. Longer headed dogs, on the other hand, with their visual streak, can have a better (wider) picture of the obstacle, where they can notice the endpoint of the fence easier, thus, they can choose the detour more readily in the case of an unsuitably small door.

One of our hypotheses predicted that if the dogs were given three opportunities to practice detouring before the large door was opened, they would habitually keep detouring. However, we found that dogs always preferred the large door over the detour. This gave an interesting opportunity to compare this experiment with the one of Pongrácz et al.,^37^, where dogs preferred the longer detour over the shortcut through the door, if they had the opportunity to perform three detour-only trials at first. A possible explanation for this could be that in the earlier study, these detours were preceded by human demonstration, therefore, one may assume that dogs tend to stick to solutions that they learned socially from a human demonstrator^30,37,38^. As the present experiment was always based on asocial (trial-and-error) learning, we can assume that dogs easily switch to newly emerging and body-size appropriate shortcuts against earlier followed, but suboptimal solutions, that haven't been reinforced by social learning. Another difference between the present and the earlier study I the topography of the obstacle that the dogs had to detour around. Here we used a straight fence, where dogs approached the door in a right angle, while Pongrácz et al.,^37^ utilized the V-shaped fence where dogs approached the door in a sharp angle. Although in the earlier study the doors were always large enough for the dogs to get through, because of approaching them in a sharp angle probably made them less conspicuous for the dogs, thus enhanced the probability of a detour in that study. In Group 3, the small door was open, therefore eventually almost every dog opted for the detour. However, longer headed dogs, due to their wider field of vision (i.e., visual streak in their retina), may recognize the endpoint of the fence easier, thus they can perform the detour more readily than the short headed dogs. Although the difference was not significant in Group 1, the same tendency indicates that those longer headed dogs that became 'habitual detour makers' during the first three trials with closed doors, again, can decide to use the detour option faster, than the short headed dogs that mostly focus ahead and tend to choose what is in front of them.

We hypothesized that detour latencies would be associated with the dogs' preference for the type of solution. Accordingly, if dogs rely on their body-size representation, in the case of the small open door, the detour will always be the optimal solution. Contrarily, the large open door would always be the better solution against the detour. Our results fit well with this hypothesis. In the case of the first trial with the small open door, dogs needed longer time to decide that this opening is not suitable for them, and it also took longer time to choose detour in this case. However, across the repeated small door trials they became more effective. In the case of the large open door, only a small proportion of dogs chose the suboptimal detour. Interestingly, these were present all along the trials, and it is also possible that those who chose to detour against the large door, did hesitate how to choose before each trial: the door (shortcut) or (the longer but familiar) detour. As we learned from our earlier experiment^37^, dogs may prefer to rely on the successful solution against a new one, therefore, it is not surprising that (although in small numbers), here we found dogs that remained with the earlier successful solution. This type of behavior can be regarded as a perseverative error^39^. As in the case of the large open door, detour is regarded as a suboptimal solution.

By comparing the detour latencies between the small and large open door trials, we could support our theory regarding how dogs decide between either the optimal (large door) vs. suboptimal (detour) solution types; or between erroneous (small door) vs. optimal (detour) solutions. As they performed the detour faster in each trial when the small door was open, than in the trials where the large door was open, we can assume that based on their body size representation, they could decide easier between the two options when the small (clearly erroneous) door was open. In the case of the large door, it took longer for them to decide between two solutions that were both viable solutions, only not equally optimal.

Similarly, the latencies of approaching the doors showed that dogs decided what to do based on their body size representation. In the repeated large door trials, they approached the door faster as the trials progressed, while they rarely approached the small door and even this proportion became somewhat smaller throughout the repeated trials.

Navigating around or through transparent fence obstacles is a difficult task for dogs^30,37,40,41^, and we found earlier that dogs look back at the owner more frequently if they have more problems with performing a detour. Therefore we hypothesized that here we would also find more human-directed gazing depending on the level of difficulty of the given type of solution. Our results confirmed the prediction. Dogs looked at the humans (owner or experimenter) least often when they had access to the large door (most optimal solution), and they gazed at the humans most frequently when they had to perform a detour (closed doors—the most problematic task). Small doors could represent a moderately problematic task, because dogs can decide rather easily that this is not a viable solution (based on body-awareness), thus they more easily decide to detour. The visible treat behind the fence (in the case of the closed doors) creates a 'visual magnet' effect, which elicits an approach reaction from dogs^42^. This makes it clear why detour is a more difficult task than going through the door: when the door is closed, dogs have to inhibit their automatic motor reaction to directly approach the food^43^. An alternative or additional explanation could be that trials with the closed doors preceded the open door trials, therefore the dogs were less experienced with the task and they found it more difficult at the beginning.

Although looking at the owner and at the experimenter suggested the same order of difficulty among the various types of solutions, dogs looked at the owner less often than at the experimenter. This could be caused by the topographic arrangement of our test (the experimenter was on the side of the treat, while the owner stood behind the dog). Additionally, earlier we found that an (unknown) experimenter was just as effective as a demonstrator for dogs, as the owner was^30^, thus we can assume that dogs would also rely readily on the experimenter as social support (see also^44^). Earlier, we showed that short headed dogs approached more often the small open door, which is an erroneous decision. We also found that shorter headed dogs looked at the experimenter more often in Group 3 (small door is preceded by only one trial with closed doors), thus we can assume that in the case of such a decision, these dogs realize that they cannot solve the problem by going through the too small opening, and this (i.e., the more difficult situation), elicits more frequent gazing at the human. The higher frequency of gazing at the experimenter in the shorter headed dogs could also be enhanced by the stronger inclination of these dogs to look at a humans' face^45^.

We found that dogs looked least frequently to humans when confronted with the large open door—this fits well with the prediction that this is the easiest solution for them, and also the shortcut that is the fastest to decide based on body representation that will fit the size of the dog. Dogs, however, looked at the small door and towards the closed door equally as often. This may indicate that these options proved to be equally hard decisions for the dogs: looking towards the closed doors could indicate their intention of getting to the treat while hesitating to make a detour. Frequent looking at the small door can also indicate that the dogs try to estimate whether they would fit through. We also found that long headed dogs looked at all of the doors less frequently than short headed dogs did. Similarly to the previous results that included an effect of CI, this finding fits well to the picture where short headed dogs would rather focus on the target right ahead of them (thus they notice the open doors more easily), while longer headed dogs would have easier visual access to the wider surroundings.

## Conclusion

Our previous research has already shown that dogs are suitable subjects for testing various modules of self-awareness^8,10,11^. Dogs live in an increasingly complex and variable anthropogenic niche since their domestication, where they encounter spatial problems requiring body-size awareness on a daily basis. In theory, animals in a simpler and more stable environment could rely on simpler mental mechanisms to negotiate spatial problems such as memorizing each obstacle (the so-called snapshot method,^46^). In our experiment, we successfully showed that dogs flexibly rely on their body-size awareness in a novel spatial problem-solving task that offered various types of solutions. In cognitive ethology, it is essential to design experiments that take into account the target species' evolutionary history and original environment. This provides a unique opportunity to investigate and understand the development and function of self-awareness modules and the complex entirety of a given species' self-representation.

Our experimental device and setup were completely novel to the dogs in our test. Therefore, we can exclude the option that they simply remembered these obstacles, or memorized whether these doors would be suitable or not for them. Only in this case would it be reasonable to assume that they solved the problem without relying on their body-size awareness. Ideally, we should have provided each dog with a small door that would provide just a big-enough opening for them to go through. However, our earlier study^8^ showed that setting the just-big-enough door requires close adjustment to the given dog's size, and as with the present metal-and-mesh device it was not feasible to achieve, we opted for the fixed-size small door.

In the future, our next effort will be to compare dogs' problem-solving behavior in a similar multi-choice obstacle paradigm, utilizing both body size awareness and social learning from a human demonstrator.

## Materials and methods (based on^37^)

### Ethical approval for animals used in the study

The study was approved by the Eötvös Loránd University (‘Animal Welfare Committee, Eötvös Loránd University, Budapest’), who checked and accepted the experimental method (Ref. no.: PE/EA/2021-5/2017). All tests were performed in accordance with the Hungarian regulations on animal experimentation and the Guidelines for the use of animals in research described by the Association for the Study Animal Behaviour (ASAB). Informed consent was obtained from the dog owners before involving their dogs in the study. Before the tests, dog owners were informed about the procedure, however we only explained the aim of the study after the test, to avoid unwanted cueing of the subjects.

In our experiments we did not test human participants, we did not collect the dog owners’ data, and thus no human ethical approval was needed.

### Subjects

We tested N = 77 family dogs that were more than 1 year old, regardless of their sex or breed. The only inclusion criterion was that their height at the withers had to be between 50 and 65 cm. Table 1 shows the basic details of the subjects and their group-assignments.

**Table 1:** List of the subjects, including their breed, height at the withers, cephalic index, and group assignments.

Dogs' ID	Breed	Height (cm)	Cephalic index	Group
1	Am. Staffordshire Terrier	50	75.78	Group 1
2	Border Collie	50.5	62.50	Group 1
3	Mongrel	51	63.04	Group 1
4	Mongrel	51	66.63	Group 1
5	Labrador Retriever	52	58.41	Group 1
6	Mongrel	54	46.23	Group 1
7	Mongrel	55	51.86	Group 1
8	Border Collie	56	49.45	Group 1
9	Whippet	56		Group 1
10	Labrador Retriever	57	55.03	Group 1
11	Mongrel	57	59.27	Group 1
12	Labrador Retriever	57	51.13	Group 1
13	Mongrel	57	56.09	Group 1
14	Labrador Retriever	60	47.41	Group 1
15	German Shepherd Dog	62	47.51	Group 1
16	Akita Inu	63	57.00	Group 1
17	Mongrel	64		Group 1
18	German Shepherd Dog	64	52.04	Group 1
19	Transylvanian Hound	65	49.39	Group 1
20	German Shepherd Dog	65	50.10	Group 1
21	Hungarian Greyhound	66	46.33	Group 1
22	Mongrel	67		Group 1
23	Mongrel	50	61.98	Group 2
24	Border Collie	51	50.47	Group 2
25	Mongrel	51	55.35	Group 2
26	Mongrel	52	61.99	Group 2
27	Mongrel	53	59.08	Group 2
28	Mongrel	53		Group 2
29	Golden Retriever	53	55.95	Group 2
30	Border Collie	55	50.75	Group 2
31	Mongrel	56	54.26	Group 2
32	Staffordshire Terrier	57	75.78	Group 2
33	Mongrel	57	50.66	Group 2
34	Mongrel	57	60.03	Group 2
35	Pitbull Terrier	57	57.93	Group 2
36	Yakutian Laika	57	62.14	Group 2
37	Mongrel	58	58.95	Group 2
38	Malinois	58		Group 2
39	Mongrel	58.5	56.04	Group 2
40	Mongrel	59	45.08	Group 2
41	Vizsla	60	50.93	Group 2
42	Mongrel	61		Group 2
43	Labrador Retriever	61	55.40	Group 2
44	Golden Retriever	61		Group 2
45	Kuvasz	64	48.23	Group 2
46	Mongrel	50		Group 3
47	Mongrel	50	54.51	Group 3
48	Mongrel	51	47.89	Group 3
49	Kelpie	52	66.31	Group 3
50	Collie	52	48.18	Group 3
51	Vizsla	54	50.93	Group 3
52	Pitbull Terrier	54		Group 3
53	Golden Retriever	56	55.53	Group 3
54	Vizsla	56	48.88	Group 3
55	Mongrel	56	51.80	Group 3
56	Samoyed	57	64.41	Group 3
57	Rottweiler	57.5	65.38	Group 3
58	Standard Poodle	58		Group 3
59	Dogo Argentino	58	63.23	Group 3
60	German Shorthaired Pointer	58.5	50.59	Group 3
61	Golden Retriever	60	55.53	Group 3
62	Mongrel	61	50.26	Group 3
63	Bernese Mountain Dog	61	60.28	Group 3
64	Siberian Husky	61.5	57.13	Group 3
65	Mongrel	62		Group 3
66	Mongrel	64	49.07	Group 3
67	Mongrel	65	57.27	Group 3
68	Irish Setter	65	42.17	Group 3

Cephalic index values are missing in those cases where the dog owners did not supply photographs of their dog’s head.

### Experimental setting

**Figure 7:**
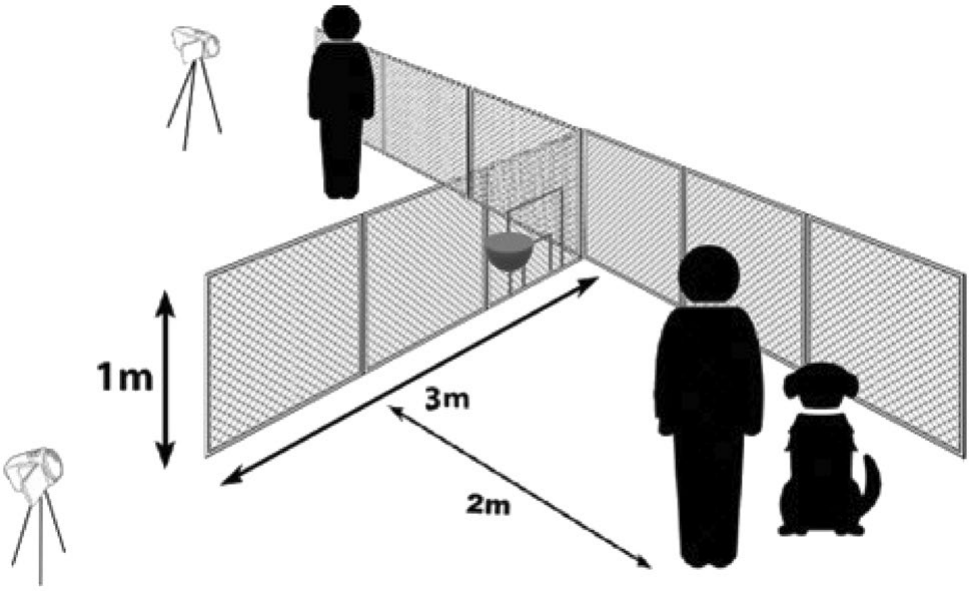
Experimental setup. The short experimental fence was attached to the long fence that served as a property border (on the drawing, only a short section is depicted from the long fence). In the first segment of the experimental fence, the small and large doors are visible, both are in closed position. The relative positions of the experimenter, owner and dog are indicated as they were at the beginning of each trial. The bowl behind the doors shows the position where the reward was placed for the dogs. We recorded the trials with two cameras, their positions are also indicated.

The tests took place on a plain grassy outdoor area at the Eötvös Loránd University's Campus. The experimental setup is illustrated in Fig. 7. For the test, a 1 m high and 3 m long fence made of thin, transparent wire mesh with a hole-diameter of 20 mm was used, attached to a steel frame. One end of the experimental fence, was fastened to the fence along the property border, creating a 90° angle. The long fence at the property border could not be detoured around. The short fence was set up by pushing the pegs protruding from the bottom edge of the frame into the ground. The frame of the fence prevented the dogs from digging under it. Two swing-doors (a small and a large one) were placed on the shorter fence, inside each other, in the middle of the first 1-m long section. These doors were constructed in a 'door in the door' fashion, and closed on the lower edge of the fence. The doors could be fixed in either an open or a closed position. The large door had a 68 × 68 cm opening, and the small one had a 21 × 21 cm opening. The latter was too small for the subjects to go through. We calculated the door sizes based on our previous experiments^8^. Based on these earlier results, although dogs noticed and approached small openings (vertically a third of the height at the withers of the dog), they decided not to go through it later. We also found that dogs, without hesitation, went through an opening of the exact vertical size of their height at the withers.

To recruit enough subjects, we tested dogs whose height at the withers fell between 50 and 65 cm. We calculated the size of the small door based on the size of the smallest dog in the range, and the large door was sized for the largest dog. Before each trial, the owner positioned the dog at the starting point, which was 2 m from the fence. This meant that the detouring route would be approximately 7 m long, compared to the 2 m long shorter one through the opening.

The tests were recorded with two hand-held cameras (Sony, FDR-AX33), both mounted on tripods. One was placed 5 m from the intersection of the two fences, in line with the short fence, while the other was placed behind the shorter fence, 5 m away from it and facing the owner and the dog.

### General procedure

Before the test, the dogs were kept on a leash at the testing site, and they were not allowed to explore the fence. The Experimenter (E) measured the dog's height at the withers while the dog stood still and the Owner (O) was asked to assist when necessary. The measuring tape was held in a taut, straight line while the measurement was taken. Before the experiment, as a warm-up phase, the E gave a treat (a piece of sausage placed on the plate) to familiarize the dog with the plate used during the test.

Before the actual trial began, we asked the O to stand on the starting point with the dog, while both of them facing away from the short fence. Then, the E walked behind the short fence and signaled the O to turn back with the dog towards her. The E showed the plate with a treat and loudly called the dog (by saying once the dog's name and the word "Look!"). At the moment the E put down the plate and stood up, the owner released the dog. The O was allowed to encourage the dog to get the reward, but he/she was asked to stay at the starting point and not use any commands or cues, such as pointing with a hand, to guide the dog around or choose the opening. The dog had 1 min to reach the treat either by detouring the fence or passing through the opening. If the dog managed to get the treat, or after 1 min elapsed, the trial ended, and the O called the dog back. Between the trials, the O positioned the dog at the starting point and was asked to turn around, preventing the dog from looking at the fence for 30 s, giving the E time to set the opening (i.e., open and fix one of the doors) when it was necessary. When the 30 s elapsed, the E indicated to turn back, and the next trial began.

### Exclusions

Seven subjects were excluded from testing because we could not motivate them with food (2 German Shepherd Dogs, 1 Dogue de Bordeaux, 1 Golden Retriever, 1 English setter, 1 Galgo, 1 mongrel). These dogs either did not eat the offered food during the warm-up phase or did not leave their owner's side in three consecutive trials during the test. One dog was excluded because its owner used additional food rewards during the test against the previously set rules. Finally, another dog was excluded because, upon their arrival at the testing site, the owner let it off-leash to explore the testing equipment. Altogether 9 dogs were excluded, resulting in statistics being run on N = 68 subjects.

### Experimental groups

We filled the experimental groups in a parallel manner, and the dogs were randomly sorted into three groups. Each dog was tested only in one condition. We wanted to investigate how dogs potentially adjust their behavior when the shorter route becomes available. Therefore, each condition started with a trial where the door was closed, and detouring the fence was the only solution. In Groups 2 and 3, where only the first trial was with closed doors, we ensured that each participant had experience with one successful detour. Thus, if the dog did not succeed at first with the detour, we repeated this trial a maximum of two more times. The dog was excluded if it could not perform a successful detour during three trials in these groups (which did not happen). In Groups 2 and 3, the first trial was followed by three trials when either the large or the small door was open. We were also interested in how flexibly dogs change their problem-solving behavior after they get more experienced in detouring the fence. Thus, in Group 1, dogs had the opportunity to detour the fence three times with the door closed before the three trials with the large door opened. In each group, after the three open-door trials, we added three more trials with the other door open. The trial order in each group is shown in Table 2.

**Table 2:** The three experimental groups, with the number of subjects tested in each.

Group 1 N = 22	Trial C1	Trial C2	Trial C3	Trial L1	Trial L2	Trial L3	Trial S1	Trial S2	Trial S3
Group 2 N = 23	Trial C1	Trial L1	Trial L2	Trial L3	Trial S1	Trial S2	Trial S3	–	–
Group 3 N = 23	Trial C1	Trial S1	Trial S2	Trial S3	Trial L1	Trial L2	Trial L3	–	–

Trials with the corresponding door configuration are indicated.

*C* closed door, *L* large door is open, *S* small door is open.

### Cephalic index measurement

We measured the dog's cephalic index from photographs that were provided by the owners upon request. The cephalic index value was measured using the rule function of the GIMP image editing program v2.2.13. (http://www.gimp.org/) from photographs taken perpendicular to the top of the skull. The skull width was measured between the two zygomatic arches, while the length was measured between the top of the nose and the occipital protuberance. The index was calculated as the ratio of the maximum width of the head multiplied by 100 divided by the head's maximum length.

### Data analysis

The behavioral coding of the video footage was performed using Solomon Coder (beta 17.03.22 copyright by András Péter). We analyzed the route choice (detour or opening) as a binary variable and the latencies of detouring the fence (measured from the dog’s departure from the starting point until the dog reached the target by going behind the fence by detouring it (s)) and approaching the opening (measured from the dog’s departure from the starting point, until the dog puts its nose into the opening (s)), as well as gazing towards the door, Owner, and Experimenter as frequencies. To check the reliability of the coding method, an independent observer coded 15 randomly chosen videos. The latency and frequency data were analyzed using Spearman's Rho-correlation. Based on the analysis, our coding procedure was reliable (detour latency: rs = 0.997; p < 0.001; reach opening latency: rs = 0.974; p < 0.001, passing-through attempts: rs = 0.967; p < 0.001, gazing: rs = 0.997; p < 0.001).

### Statistical analysis

The statistical analyses were done in R environment^47^ using RStudio^48^. The binary variables were analyzed with generalized linear mixed model (GzLMM) using binomial distributionfrequencies using Poisson distribution with log link (lme4 package, glmer function) and for the latencies, we used Mixed Effects Cox Regression (coxme package, coxme function). All models had the dog ID included as random intercept, and run with maximum likelihood estimation using Laplace Approximation. In the binomial models we used a combination of backwards and forward model selection approach: first we tested whether the Groups differed in their choice depending on the available door size in their first encounters with them (Size + Group + Size:Group) and excluded step-wise non-significant effects based on AIC value changes (drop1 function). Then in two separate models we included CI and height and their interactions, and again tested with LR test if their inclusion improves the model or not.

In the case of Poisson models and cox regressions, initial models contained main effects (Size, Group, Trial, and CI) and their interactions, and we applied backwards model selection based on AIC values (anova in coxme models and drop1 in Poisson models). The model with the lowest AIC value was kept, and we considered a model better if the δ AIC value was equal or greater than 2.

In the case of pairwise comparisons, we ran Tukey post hoc tests (emmeans package). We always reported the results of the final models.

### Supplementary Information

Supplementary Information 1.

Supplementary Information 2.

Supplementary Figures.

## Data Availability

All data generated or analyzed during this study are included in this published article [and its supplementary information file S1: Supplement_RawData_Pongraczetal_Door_or_detour.xlsx].
