# Supplementary material for: Body size awareness matters when dogs decide whether to detour an obstacle or opt for a shortcut
Source: Sci Rep. 2023 Oct 19;13:17899. doi: 10.1038/s41598-023-45241-w (PMC10587091; doi:10.1038/s41598-023-45241-w)
Supplement: Supplementary file 3 — Supplementary Figures. [file 41598_2023_45241_MOESM3_ESM.docx]

**Supplementary material - Figures**


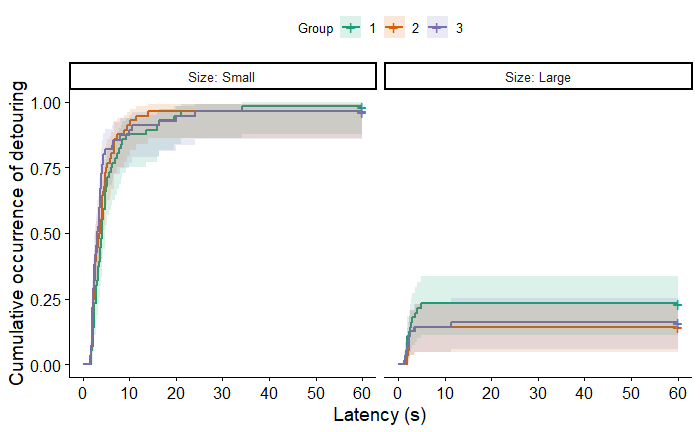


Supplementary Figure 1: Proportion of dogs performing a detour with a given latency in the case of the three trials with open small or large doors. Shading: ± 95% CI.


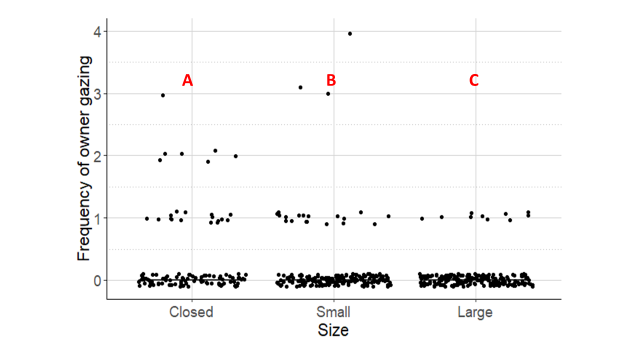


Supplementary Figure 2: Frequency of looking at the Owner. Different letters above the boxplots mark the significantly differing groups.


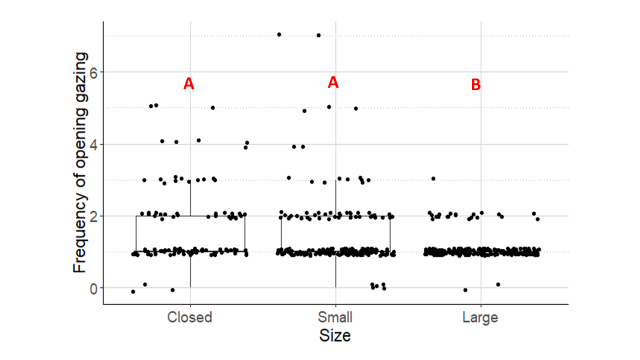


Supplementary Figure 3: Frequency of looking at the door. Different letters above the boxplots mark the significantly differing groups.


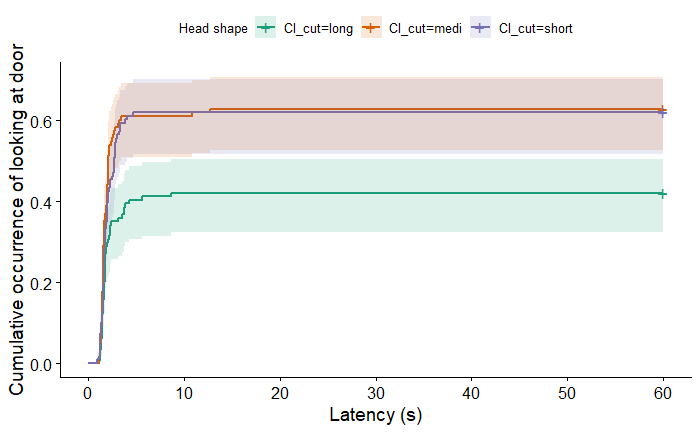


Supplementary Figure 4: Proportion of dogs at a given latency who looked at the doors (independently of door size). In order to visualize the results, the otherwise continuous CI score was divided at the 33rd and 67th percentiles. Different colors mark different lengths of head (red: long, green: meso, blue: short). Shading: ± 95% CI.
